# Quantitative analysis of signaling responses during mouse primordial germ cell specification

**DOI:** 10.1242/bio.058741

**Published:** 2021-05-07

**Authors:** Sophie M. Morgani, Anna-Katerina Hadjantonakis

**Affiliations:** Developmental Biology Program, Sloan Kettering Institute, Memorial Sloan Kettering Cancer Center, New York, NY 10065, USA

**Keywords:** BMP, MAPK, WNT, Mouse embryo, Primordial germ cell

## Abstract

During early mammalian development, the pluripotent cells of the embryo are exposed to a combination of signals that drive exit from pluripotency and germ layer differentiation. At the same time, a small population of pluripotent cells give rise to the primordial germ cells (PGCs), the precursors of the sperm and egg, which pass on heritable genetic information to the next generation. Despite the importance of PGCs, it remains unclear how they are first segregated from the soma, and if this involves distinct responses to their signaling environment. To investigate this question, we mapped BMP, MAPK and WNT signaling responses over time in PGCs and their surrounding niche *in vitro* and *in vivo* at single-cell resolution. We showed that, in the mouse embryo, early PGCs exhibit lower BMP and MAPK responses compared to neighboring extraembryonic mesoderm cells, suggesting the emergence of distinct signaling regulatory mechanisms in the germline versus soma. In contrast, PGCs and somatic cells responded comparably to WNT, indicating that this signal alone is not sufficient to promote somatic differentiation. Finally, we investigated the requirement of a BMP response for these cell fate decisions. We found that cell lines with a mutation in the BMP receptor (*Bmpr1a*^−/−^), which exhibit an impaired BMP signaling response, can efficiently generate PGC-like cells revealing that canonical BMP signaling is not cell autonomously required to direct PGC-like differentiation.

## Abstract (precis)

**Summary:** A subpopulation of pluripotent cells of the embryo give rise to the primordial germ cells (PGCs), the precursors of the sperm and egg, which pass on heritable genetic information to the next generation. To determine how PGCs are first segregated from the soma, we investigated BMP, MAPK and WNT signaling over time in PGCs and their surrounding niche *in vitro* and *in vivo* at single-cell resolution.

## INTRODUCTION

Primordial germ cells (PGCs) are the embryonic precursors of the sperm and egg, required to pass on heritable genetic information to the next generation. Defects in PGC production result in infertility while transformed or incorrectly positioned PGCs may give rise to germ cell tumors (Pierce et al., 2018; Stevens, 1967; 1980; Giuliano et al., 2006). Thus, delineating the mechanisms that control PGC formation is essential to understand both development and disease.

In mouse, PGCs arise during early development when the pluripotent epiblast of the embryo is exposed to a myriad of signals (Morgani and Hadjantonakis, 2020) that direct most cells to adopt a somatic fate and only around 40 cells to become PGCs (Magnusdottir et al., 2013; Ohinata et al., 2005; Grabole et al., 2013). While many of the signals that regulate PGC specification have been elucidated (Ohinata et al., 2009; Saitou and Yamaji, 2010; Senft et al., 2019), it is unclear how germline and soma identities emerge within a common signaling environment, how PGCs and their niche respond to these signals, and how signaling responses change over time.

To address this, we quantitatively analyzed the response of individual presumptive PGCs within the allantois and surrounding non-PGCs to key signals present within the embryo. We showed that PGCs displayed significantly lower Bone Morphogenetic Protein (BMP) and Mitogen-Activated Protein Kinase (MAPK) responses compared to non-PGCs, indicating cell type-specific modes of pathway regulation. In contrast, PGCs and non-PGCs responded comparably to WNT, demonstrating that PGCs are not refractory to all signals. Finally, we showed that embryonic stem cells (ESCs) defective in their canonical BMP signaling response efficiently generated PGC-like cells (PGCLCs). Hence, a canonical BMP response is not cell autonomously or non-cell autonomously required for PGCLC differentiation *in vitro*.

## RESULTS AND DISCUSSION

### Quantitative analysis of signaling responses during mouse PGCLC specification

**Fig. 1.**
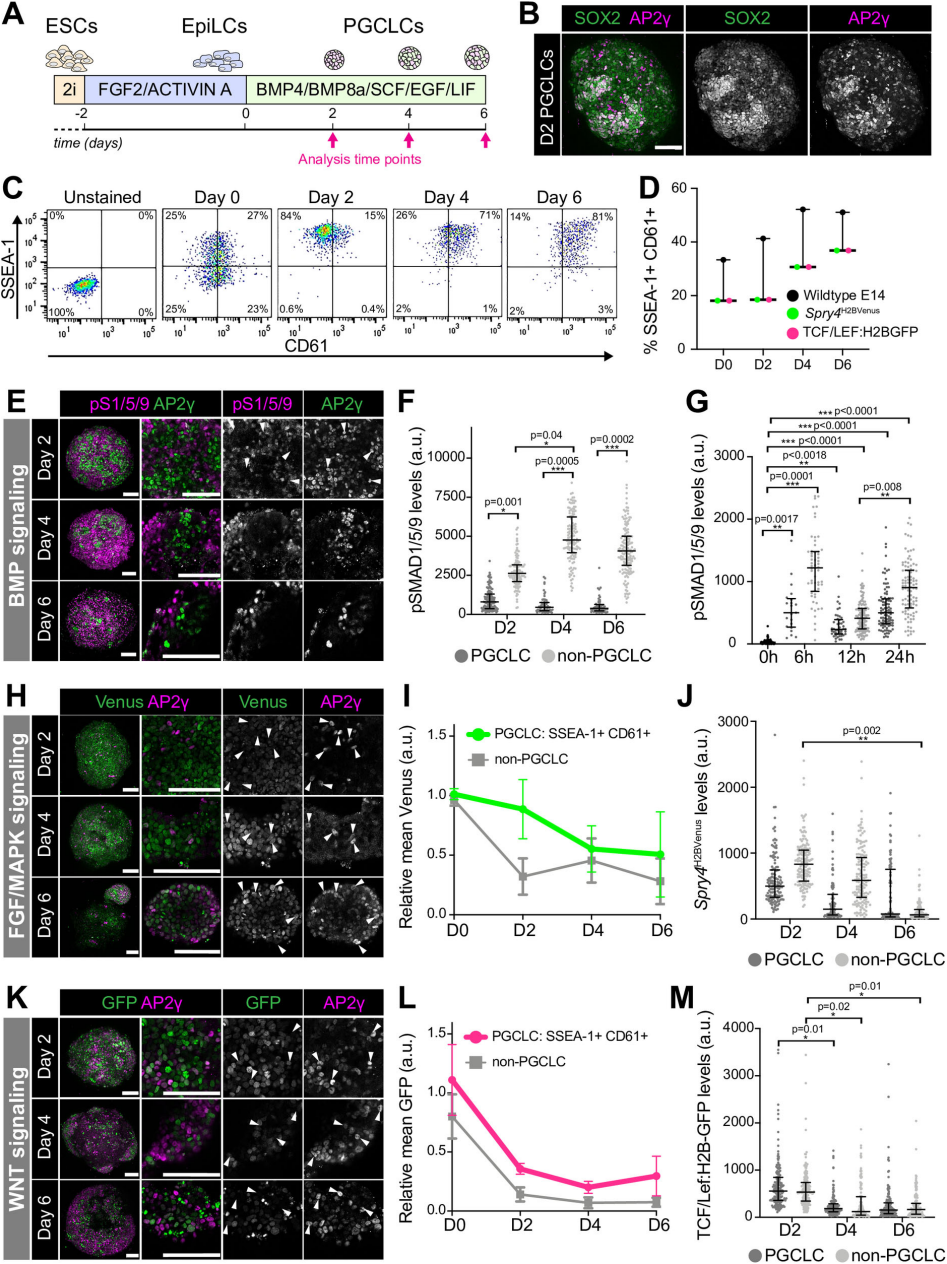
**Quantitative analysis PGCLC signaling responses.** (A) Diagram depicting PGCLC differentiation protocol (Hayashi et al., 2011). (B) Confocal maximum intensity projection (MIP) of a Day 2 (D2) PGCLC aggregate. Scale bars: 100 μm. (C) Flow cytometry data from PGCLC differentiation. SSEA-1+ CD61+ cells represent PGCLCs. (D) Percentage of SSEA-1+ CD61+ PGCLCs over time. Each point represents an independent experiment (*n*=6) performed with four cell lines, represented as median and interquartile range. (E,H,K) Confocal MIPs of PGCLC aggregates at day 2, 4, and 6. Sb, 100 μm. (E) Aggregates immunostained for AP2γ (PGCLCs) and phosphorylated SMAD1/5/9 (pS1/5/9), a readout of BMP signaling response. (H) PGCLC differentiation of *Spry4*^H2BVenus^ reporter ESCs, that read out FGF/MAPK signaling activity. (K) PGCLC differentiation of TCF/Lef:H2B-GFP reporter ESCs, which read out WNT signaling activity. (F,J,M) Quantitative immunofluorescence of signaling responses in PGCLCs (AP2γ+) and non-PGCLCs (AP2γ−) in three cell aggregates/time point/cell line. Each point represents a single cell. Data shown as median and interquartile range. Student's *t*-test was performed on average fluorescence level per aggregate. (G) Quantitative immunofluorescence of signaling responses in PGCLCs (AP2γ+) and non-PGCLCs (AP2γ−) at early differentiation time points. Each point represents a single cell. Data shown as median and interquartile range. Student's *t*-test was performed on average fluorescence level per aggregate (12 h *n*=4, 24 h *n*=3). At 0 and 6 h time points, cells had not yet aggregated so statistics were performed on average fluorescence per field of view (6 h *n*=3, 0 h *n*=6). (I,L) Relative mean *Spry4*^H2BVenus^ (H) and TCF/Lef:H2B-GFP (K) fluorescence analyzed by flow cytometry. Data represented as mean and standard deviation and shown relative to mean fluorescence across all populations at day 0. *n*=3 experiments.

Under defined *in vitro* conditions (Fig. 1A) (Hayashi et al., 2011), mouse ESCs generate PGCLCs that give rise to functional germ cells (Hayashi et al., 2011; 2012; Hikabe et al., 2016; Ishikura et al., 2016). We generated PGCLCs, as described (Hayashi et al., 2011), and identified by the co-expression of SOX2 and AP2γ (Fig. 1B), and cell surface markers SSEA-1 and CD61 (Fig. 1C,D) (Hayashi and Saitou, 2013). PGCLC aggregates displayed widespread SOX2 expression while AP2γ was expressed in only a subset of cells (Fig. 1B). Thus, we analyzed signaling responses in SOX2+ AP2γ+ cells, considered to be PGCLCs, as well as surrounding AP2γ− non-PGCLCs.

BMP signaling plays a critical role in germ cell specification. Mutations in genes encoding *Bmp4*, *Bmp8*, and *Bmp2*, and the downstream signaling effectors, *Smad1* and *Smad5*, result in a loss or significant reduction in PGC number (Chang and Matzuk, 2001; Hayashi et al., 2002; Lawson et al., 1999; Tremblay et al., 2001; Ying et al., 2000; Ying and Zhao, 2001). However, these mutants also display defects in allantois formation and hence, in the absence of PGC-specific Cre drivers to generate conditional knockouts, it has been difficult to tease apart the requirement of BMP signaling for extraembryonic mesoderm versus PGC specification. Moreover, neither PGCLCs *in vitro* nor PGCs *in vivo* exhibit a canonical BMP signaling response (Senft et al., 2019; Dudley et al., 2007), further confounding this issue. Nevertheless, BMP responses have not been quantitatively analyzed at single-cell resolution hence it is unclear whether a fraction of PGCs may respond or if an earlier, transient response occurs. To investigate this, we quantified protein levels of the downstream effector of BMP signaling, phosphorylated (p) SMAD1/5/9, in individual nuclei at days 2, 4, and 6 of PGCLC differentiation. SOX2+ AP2γ+ PGCLCs displayed significantly lower nuclear pSMAD1/5/9 than AP2γ− non-PGCLCs (Fig. 1E,F). Indeed, we did not identify any PGCLCs with clear nuclear-localized pSMAD1/5/9 (Fig. 1E,F). Furthermore, while the BMP signaling response increased in non-PGCLCs over time, it remained low in PGCLCs (Fig. 1F). Thus, at this temporal resolution, we did not observe BMP-responsive PGCLCs. In order to determine whether AP2γ+ cells exhibited an early, transient BMP response during PGCLC differentiation, we proceeded to analyze cells at 0 h and 6, 12 and 24 h following cytokine addition. We found that AP2γ+ cells showed a small but significant increase in nuclear-localized pSMAD1/5/9 at 6, 12 and 24 versus 0 h (Fig. 1G), indicating that these cells do respond to BMP, albeit at low levels. BMP signaling activity increased in AP2γ− but not AP2γ+ cells over time (Fig. 1G).

We then asked whether PGCLCs lack responses to other critical signals present within the mouse embryo at this time. FGFs are expressed during PGC specification and are necessary for somatic germ layer specification, the gastrulation EMT, and concomitant cell migration (Yamaguchi et al., 1994; Ciruna and Rossant, 2001; Deng et al., 1994). Additionally, both FGF and EGF that activate the MAPK pathway, are added exogenously to PGCLC culture medium (Fig. 1A). To analyze the MAPK response, we used *Spry4*^H2BVenus^ ESCs, which harbor a fluorescent reporter in the endogenous locus of *Sprouty4* (*Spry4*), an early pathway target (Morgani et al., 2018a). Venus expression was observed throughout PGCLC aggregates at all stages of differentiation (Fig. 1H). In contrast to the gradually increasing BMP response in non-PGCLCs, there was a reduction in the MAPK response over time (Fig. 1I,J). Quantitative immunofluorescence revealed no significant difference in the MAPK response in PGCLCs versus non-PGCLCs (Fig. 1J), although Venus levels were slightly lower in AP2γ+ versus AP2γ− cells (Fig. 1J).

WNT signaling is required to specify both somatic (Barrow et al., 2007; Liu et al., 1999; Haegel et al., 1995; Kelly et al., 2004) and germ cell (Ohinata et al., 2009; Aramaki et al., 2013) fates. Here we used TCF/Lef:H2B-GFP reporter ESCs to read out the WNT signaling response (Ferrer-Vaquer et al., 2010) during PGCLC differentiation. Although recombinant WNT is not added exogenously to PGCLC medium, TCF/Lef:H2B-GFP was heterogeneously expressed in cell aggregates (Fig. 1K), signifying the presence of endogenous WNT ligands. There was no difference in the WNT response in PGCLCs compared to non-PGCLCs (Fig. 1L,M) and therefore, PGCLCs are not refractory to all differentiation-inducing signals. Previous studies suggest that WNT drives the initial exit from pluripotency but a subset of its targets must subsequently be repressed in PGC-fated cells to block the somatic trajectory (Aramaki et al., 2013). Consistent with this, the WNT response decreased during PGCLC differentiation (Fig. 1L,M). Thus, PGCLCs initially show a reduced BMP signaling response and, as differentiation proceeds, PGCLCs and non-PGCLCs also reduce their MAPK and WNT signaling responses.

### Quantitative analysis of signaling responses during PGC specification *in vivo*

**Fig. 2.**
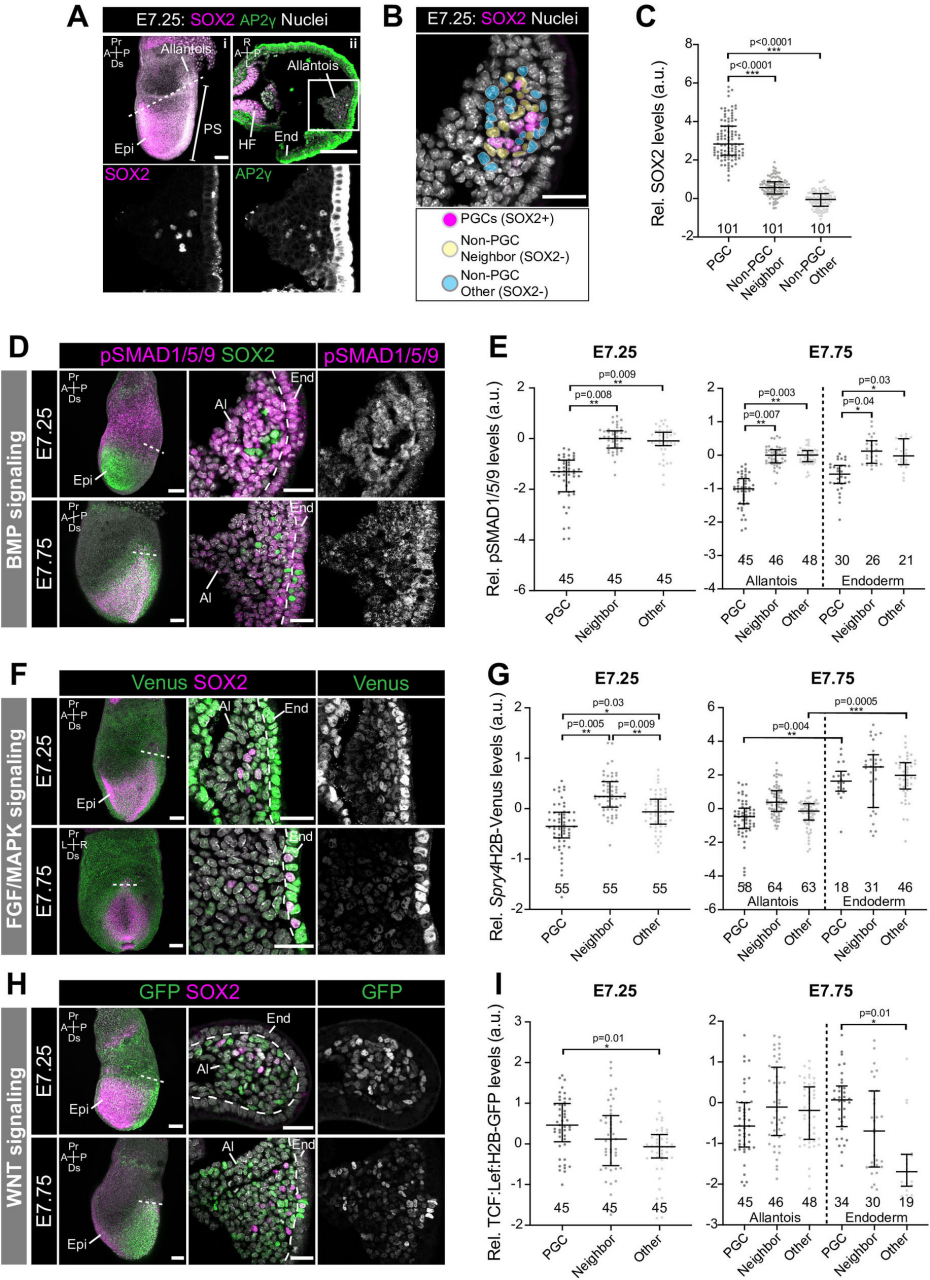
**Quantitative analysis of signaling responses during PGC specification *in vivo*.** (A) (i) Sagittal confocal optical section of an immunostained E7.25 embryo. Scale bar: 100 μm. Dashed line indicates plane of transverse section in adjacent panel. (ii) Confocal optical section of a transverse cryosection through the E7.25 allantois. Scale bar: 25 μm. Box demarcates region in higher magnification in lower panels. (B) Confocal image of a transverse section of the allantois indicating the different cell populations analyzed. Cells adjacent to PGCs (yellow) were categorized as PGC ‘Neighbors’ and non-adjacent cells within the allantois (blue) as ‘Other’ (cell populations were manually selected and pseudocolored for illustrative purposes). (C) Quantification of SOX2 levels in PGCs, Neighbors and Others within the E7.25 allantois. SOX2+ levels were used to define the PGC population. Student's *t*-test was performed on average fluorescence level in each embryo (*n*=3 embryos, number of cells indicated on graph). Each point represents a single cell. Data shown relative to average mean fluorescence in ‘Other’, non-PGCs and represented as median and interquartile range. (D,F,H) Sagittal confocal MIPs (left panels, Scale bar: 100 μm) and confocal optical sections of transverse cryosection through E7.25 and E7.75 allantois’ (Scale bar: 25 μm). Dashed line demarcates boundary between allantois and endoderm. (D) Embryos immunostained for pSMAD1/5/9. (F) *Spry4*^H2BVenus^ reporter embryos. (H) TCF/Lef:H2B-GFP reporter embryos. (E,G,I) Quantification of nuclear pSMAD1/5/9, *Spry4*^H2BVenus^, and TCF/Lef:H2B-GFP levels in PGCs, Neighbors and Other cells within the E7.25 and E7.75 allantois’. Student's *t*-test was performed on average fluorescence level per embryo (*n*=3 embryos, number of cells indicated on graph). Each point represents a single cell. Data shown relative to average mean fluorescence in ‘Other’, non-PGCs and represented as median and interquartile range. Pr, proximal; Ds, distal; A, anterior; P, posterior; L, left; R, right; Epi, epiblast; HF; headfold; PS, primitive streak; End, endoderm.

The combination, dynamics, and dose of factors provided during PGCLC differentiation *in vitro*, may not precisely recapitulate the signaling environment within the mouse embryo. Moreover, as the majority of AP2γ− non-PGCLCs expressed SOX2 (Fig. 1B), they likely represent a pluripotent EpiLC or earlier PGCLC state, and thus do not mirror the *in vivo* PGC niche that comprises extraembryonic mesoderm. Therefore, we sought to investigate signaling responses in PGCs and their niche in the embryo. Presumptive SOX2+ AP2γ+ PGCs emerge within a posteriorly-localized extraembryonic structure known as the allantois at around embryonic day (E) 7.25 (Fig. 2A) (Ginsburg et al., 1990). While a dearth of cell type-specific markers for this population has impeded genetic lineage tracing experiments, live imaging revealed that the vast majority of these SOX2+ cells migrate along the hindgut toward the gonads (McDole et al., 2018). We isolated and analyzed mouse embryos at embryonic day E7.25, when SOX2+ AP2γ+ arise within the allantois, and at E7.75, when they begin to migrate.

In contrast to PGCLC aggregates, where only a subset of SOX2+ cells expressed AP2γ, SOX2 and AP2γ expression fully overlapped at these stages *in vivo* (Fig. 2A). As AP2γ immunofluorescence resulted in high levels of non-specific staining in the endoderm on the embryo's surface (Fig. 2A), we used SOX2 to accurately identify this population. We isolated wild-type embryos, which we immunostained for pSMAD1/5/9, as well as *Spry4^H2B-Venus^*, and TCF/Lef:H2B-GFP reporter embryos and measured signaling responses in SOX2+ PGCs, and SOX2− non-PGCs that were adjacent to PGCs (categorized as ‘Neighbors’), or non-adjacent (categorized as ‘Other’) in transverse cryosections of the allantois (Fig. 2A–C). As in PGCLCs, PGCs at E7.25 and E7.75 showed significantly lower levels of nuclear-localized pSMAD1/5/9 than both neighboring and non-neighboring SOX2− cells (Fig. 2D,E). Together these data suggest that a robust canonical BMP signaling response is not required cell autonomously in specified PGCs.

*In vitro*, FGF/MAPK signaling drives the reprogramming of PGCs to an earlier state of pluripotency (Chang et al., 2020). Conversely, MAPK inhibition supports PGC differentiation (Kimura et al., 2014). Thus, FGF/MAPK signaling activity is negatively correlated with a PGC identity. In keeping with this, at E7.25, PGCs displayed a significantly lower MAPK response than non-PGCs (Fig. 2F,G). By E7.75 this difference was no longer significant (Fig. 2G), suggesting that FGF/MAPK signaling does not destabilize PGC identity at later stages of development. Endoderm-localized migratory PGCs displayed a higher MAPK response than PGCs remaining within the allantois (Fig. 2G). The MAPK response was also higher in endoderm versus allantois (extraembryonic mesoderm) cells (Fig. 2G). Therefore, as PGCs migrate towards the gonads, they are exposed to an environment that promotes elevated MAPK signaling activity, consistent with studies showing that FGF regulates germ cell migration (Chang et al., 2020; Takeuchi et al., 2005). Nevertheless, this is at odds with reports that migrating PGCs are devoid of phosphorylated ERK, a component of the MAPK pathway (Grabole et al., 2013) and hence *Spry4^H2B-Venus^* expression may be affected by additional signaling inputs, such as WNT (Katoh and Katoh, 2006).

PGCs are specified in a signaling-rich environment that instructs the majority of cells to adopt a somatic non-PGC identity. One way that PGCs might maintain their unique identity is via mechanisms that prevent them from detecting or responding to these signals. Nevertheless, while PGCs displayed reduced BMP and MAPK responses, they did respond to WNT. We previously showed that there was no difference in the WNT response in PGCLCs versus non-PGCLCs *in vitro* (Fig. 1L). However, E7.25 PGCs *in vivo* expressed higher levels of TCF/Lef:H2B-GFP than non-adjacent extraembryonic mesoderm cells (Fig. 2H,I). The significant differences in MAPK and WNT signaling responses in embryonic PGCs versus non-PGCs but not in PGCLC aggregates is presumably due to differences in the identity of non-PGC populations *in vitro* versus *in vivo*, highlighting the importance of these comparisons. At E7.75, migrating PGCs also exhibited a stronger WNT response than non-adjacent endoderm. Therefore, PGCs exhibited the strongest WNT response, followed by immediate neighbors, while non-neighboring, non-PGCs were least responsive. These data suggest that PGCs might be a source of WNT that activates autocrine and paracrine signaling in adjacent, but not more distant cells. Furthermore, these data indicate that, in the absence of robust BMP and MAPK responses, WNT signaling response does not drive somatic differentiation in PGC-fated cells.

### BMP signaling response is not required for PGCLC specification

**Fig. 3.**
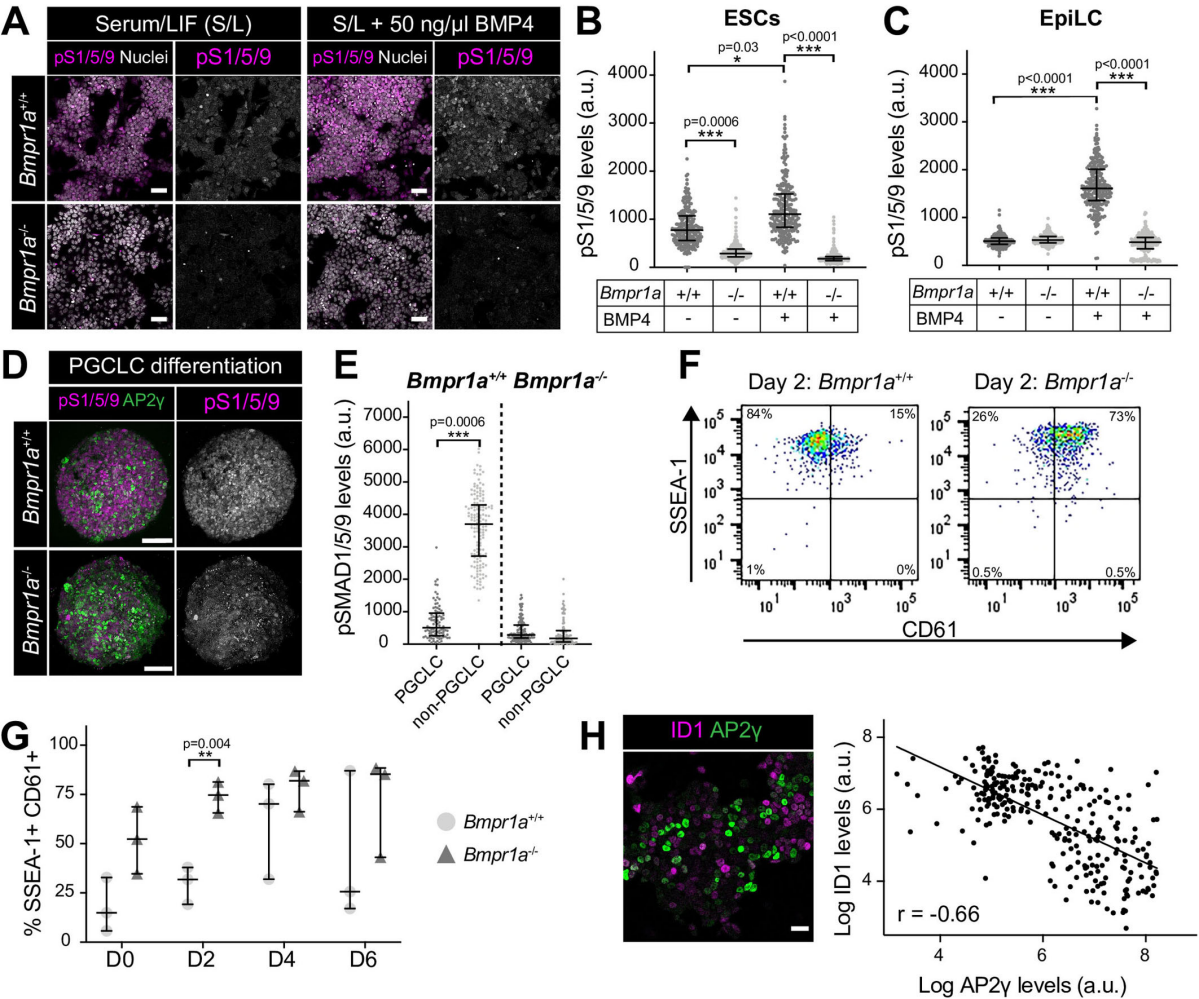
**Canonical BMP signaling is not necessary for PGCLC differentiation.** (A) Confocal optical sections of wild-type (*Bmpr1a*^+/+^) and *Bmpr1a*^−/−^ ESCs immunostained for pSMAD1/5/9 (pS1/5/9) after culture under standard conditions or after a 2-h treatment with 50 ng/ml BMP4. (B,C) Quantification of pSMAD1/5/9 levels in wild-type and *Bmpr1a*^−/−^ ESCs and epiblast-like cells (EpiLCs) from five distinct fields of view. Each point represents a single cell. Data represented as median and interquartile range. Student's *t*-test was performed on average fluorescence level in each field. *n*=2 replicates. (D) Confocal MIP of wild-type and *Bmpr1a*^−/−^ PGCLC aggregates at Day 2 (D2) of differentiation. Scale bar: 100 μm. (E) Quantification of pSMAD1/5/9 levels in wild-type and *Bmpr1a*^−/−^ PGCLC aggregates. Each point represents a single cell. Data represented as median and interquartile range. Student's *t*-test was performed on average fluorescence level per aggregate (*n*=3 aggregates). (F) Flow cytometry of wild-type and *Bmpr1a*^−/−^ aggregates at Day 2 of PGCLC differentiation. SSEA-1+ CD61+ cells represent PGCLCs. (G) Percentage of SSEA-1+ CD61+ PGCLCs during wild-type and *Bmpr1a*^−/−^ PGCLC differentiation. Each point represents an independent experiment (*n*=3). Data represented as median and interquartile range. (H) Left panel: confocal optical section of ESCs, cultured in serum and LIF, immunostained for the BMP pathway target, ID1 and the PGC marker AP2γ. Scale bar: 25 μm. Right panel: quantification of ID1 and AP2γ levels in individual cells. Quantification performed on images from five randomly selected regions. Linear regression and correlation coefficient analysis were performed (*P*<0.0001). Correlation coefficient indicated on graph. (I) Wild-type and *Bmpr1a*^−/−^ EpiLCs cells, lineage-labelled with a constitutive GFP, were mixed in equal ratios to form PGCLC aggregates. Confocal MIPs of PGCLC aggregates at day 2, 4, and 6 of differentiation. Scale bar: 100 μm.

While BMP is required for PGC specification (Chang and Matzuk, 2001; Hayashi et al., 2002; Lawson et al., 1999; Tremblay et al., 2001; Ying et al., 2000; Ying and Zhao, 2001), and BMP4 and BMP8a (500 ng/UL) are exogenously provided during PGCLC differentiation (Hayashi et al., 2011), we and others showed that neither PGCLCs nor PGCs exhibit discernable nuclear-localized pSMAD1/5/9 (Fig. 1E,F and Fig. 2C,D) (Senft et al., 2019; Dudley et al., 2007). Thus, the requirement of BMP in germ cell differentiation is still unclear. Here we leveraged *Bmpr1a*^−/−^ ESCs (Di-Gregorio et al., 2007) to ask whether a BMP signaling response is necessary for PGCLC differentiation. *Bmpr1a* is the most broadly and highly expressed BMP receptor within the pluripotent epiblast (Pijuan-Sala et al., 2019) and *Bmpr1a*^−/−^ embryos exhibit little or no nuclear pSMAD1/5/9 (Mishina et al., 1995). As previously demonstrated (Di-Gregorio et al., 2007), in contrast to wild-type ESCs, *Bmpr1a*^−/−^ ESCs did not display nuclear-localized pSMAD1/5/9 under standard serum/LIF culture conditions (Fig. 3A) or when treated with BMP4 for 2 h (Fig. 3B). Comparable observations were made with *Bmpr1a*^−/−^ EpiLCs (Fig. 3C). We then exposed *Bmpr1a*^−/−^ EpiLCs to PGCLC induction medium and showed that, likewise, *Bmpr1a*^−/−^ PGCLC aggregates did not exhibit nuclear-localized pSMAD1/5/9 (Fig. 3D,E). Despite this, cells were formed that expressed AP2γ, SSEA-1 and CD61 (Fig. 3D,F,G), suggestive of a PGCLC identity. *Bmpr1a*^−/−^ EpiLCs showed a higher percentage of SSEA-1+ CD61+ cells than wild-type EpiLCs prior to exposure to PGCLC medium, and accordingly displayed an earlier peak in this population during differentiation (Fig. 3G). Hence, we hypothesize that cells with a low BMP response may be predisposed towards a PGCLC fate. Consistent with this, we also noted an inverse correlation between the expression of the BMP pathway target Inhibitor of differentiation 1 (ID1) and the PGC marker AP2γ in wild-type ESCs (Fig. 3H).

Together, our data show that specified PGCs do not exhibit a canonical BMP signaling response (Fig. 1F and Fig. 2E) (Senft et al., 2019; Dudley et al., 2007) and early PGCLC precursors exhibit only minimal BMP signaling activity (Fig. 1G). Combined with our finding that BMP signaling defective (*Bmpr1a*^−/−^) ESCs efficiently generate PGCLCs, this suggests that either low-level BMP signaling activity is sufficient for PGC specification or alternatively that BMP signaling is not required cell autonomously for this process. As *Bmpr1a*^−/−^ PGCLC differentiation occurred in the absence of wild-type cells, the requirement for BMP is also not via paracrine interactions with BMP-responsive cells within the niche and may instead be through non-canonical SMAD-independent downstream pathways (Derynck and Zhang, 2003; Zhang, 2009). Alternatively, as perturbation of BMP signaling *in vivo* causes the epiblast to prematurely adopt a neural identity (Di-Gregorio et al., 2007), BMP may be required to initially maintain the epiblast in a PGC competent state rather than being directly involved in PGC differentiation. This role could be masked *in vitro* where ESCs are forcibly maintained in a self-renewing state using LIF or 2i small molecule inhibitors (Hayashi et al., 2011).

Here, we have shown that PGC-specific signaling responses exist for a number of pathways. However, the important question remains as to how these distinct PGCs and soma responses are regulated. To date, single-cell transcriptomic studies of mouse embryos contain only a small number of PGCs with no spatial information, prohibiting clear conclusions about the relative expression levels of signaling pathway components within PGCs and their niche. Future PGC-enriched single-cell spatial transcriptomic studies may shed light on this. Still, as signaling responses are largely regulated at a post-transcriptional level, advances in single-cell proteomic techniques or the use of quantitative time and space resolved reporters as dynamic signaling readouts may be necessary to fully address these questions.

## MATERIALS AND METHODS

### Cell culture and PGCLC *in vitro* differentiation

Cells were maintained at 37°C, at 5% CO_2_ and 90% humidity. ESC lines were routinely cultured in serum/LIF medium [Dulbecco's modified Eagle's medium (DMEM)] (Gibco, Gaithersburg, MD, USA) containing 0.1 mM non-essential amino-acids (NEAA), 2 mM glutamine and 1 mM sodium pyruvate, 100 U/ml Penicillin, 100 μg/ml Streptomycin (all from Life Technologies, Carlsbad, CA, USA), 0.1 mM 2-mercaptoethanol (Sigma-Aldrich, St. Louis, MO, USA), and 10% Fetal Calf Serum (FCS, F2442, Sigma-Aldrich) and 1000 U/ml LIF on plates coated with 0.1% gelatin, as described (Morgani et al., 2018b). The following cell lines were used in this study: E14 (129/Ola background) (Hooper et al., 1987), TCF/Lef:H2B-GFP (Ferrer-Vaquer et al., 2010), *Spry4*^H2B-Venus^ (Morgani et al., 2018a), and *Bmpr1a*^−/−^ (Di-Gregorio et al., 2007).

*In vitro* PGCLC differentiation was performed as described (Hayashi et al., 2011). Briefly, ESCs were converted to an epiblast-like (EpiLC) state by 48-h culture in N2B27 medium containing 12 ng/ml FGF2 (233-FB-025, R&D Systems) and 20 ng/mL ACTIVIN A (120-14P, Peprotech, Rocky Hills, NJ, USA) on dishes coated with 16.7 μg/mL fibronectin (FC010, Millipore). Following EpiLC conversion, cells were trypsinized to a single cell suspension and 10,000 cells/mL were resuspended in PGCLC medium, comprising GMEM (Gibco), 0.1 mM NEAA, 2 mM glutamine and 1 mM sodium pyruvate, 100 U/mL Penicillin, 100 μg/mL Streptomycin, 0.1 mM 2-mercaptoethanol, 1000 U/mL LIF, 15% Knockout serum replacement, with 500 ng/mL BMP4, 500 ng/ml BMP8a, 100 ng/mL SCF, and 50 ng/mL EGF (all from R&D Systems) and 100 μL added per well of a low adherence round bottom 96-well plate in order to form floating cell aggregates. Samples were collected for analysis at day 0 (EpiLC state), 2, 4 and 6 of differentiation. To note, as we had previously observed no difference in the efficiency of EpiLC conversion from ESCs cultured in serum/LIF compared to 2i/LIF (data not shown), our starting ESC cultures were from serum/LIF rather than 2i/LIF as previously described (Hayashi et al., 2011).

### Flow cytometry

Between 8-12 PGCLC aggregates per cell line/condition were pooled and then dissociated by incubation in TrpLE^TM^ Select Enzyme (Thermo Fisher Scientific) at 37°C for approximately 2 min. Following vigorous pipetting to form a single-cell suspension, the enzyme was neutralized with an equal volume of PGCLC medium without cytokines added. Cells were pelleted by centrifugation and then resuspended in 100 μL FACs buffer (PBS with 10% FCS) with PE-conjugated anti-CD61 (RRID:AB_313084, Biolegend, 104307, 1:200) and Alexa Fluor 647-conjugated anti-SSEA1 (RRID:AB_1210551, Thermo Fisher Scientific, 51-8813-73, 1:50) for 15 min on ice. Cells were then washed in 1 mL FACS buffer and resuspended in 200 μL FACS buffer containing 5 μg/ml Hoechst. Samples were analyzed using a BD LSR Fortessa^TM^. Flow cytometry analysis was performed using FlowJo software (BD Biosciences). Cells were first separated from debris and cell doublets removed by gating on forward (FSC) and side scatter (SSC). Subsequently, dead cells were identified based on strong Hoechst staining and were excluded from further analysis. Gating for CD61, SSEA-1 positive cells was based on unstained wild-type E14 ESCs.

### Mouse lines

Mice were housed under a 12 h light-dark cycle in a pathogen-free room in the designated MSKCC facilities. For this study we used outbred CD1 animals maintained in accordance with the guidelines of the Memorial Sloan Kettering Cancer Center (MSKCC) Institutional Animal Care and Use Committee (IACUC) under protocol number 03-12-017 (PI Hadjantonakis). Natural mating was set up in the evening and mice were checked for copulation plugs the next morning. The date of vaginal plug was estimated as E0.5. For analysis of post-implantation stages of development, embryos were isolated from deciduae and Reichert's membrane removed by microdissection before further processing.

### Immunostaining

Cell lines were immunostained as previously described (Morgani et al., 2018b). Post-implantation embryos were washed in phosphate-buffered saline (PBS), then fixed in 4% paraformaldehyde (PFA) for 15 min at room temperature (RT). Embryos were washed in PBS plus 0.1% Triton-X (PBST-T) followed by permeabilization for 30 min in PBS with 0.5% Triton-X. Embryos were then washed in PBS-T and blocked overnight at 4°C in PBS-T with 1% bovine serum albumin (BSA, Sigma-Aldrich) and 5% donkey serum (Sigma-Aldrich). The following day, embryos were transferred to the primary antibody solution (PBS-T with appropriate concentration of antibody) and incubated overnight at 4°C. The following day, embryos were washed 3×10 min in PBS-T and transferred to blocking solution at RT for a minimum of 5 h. Embryos were transferred to secondary antibody solution (PBS-T with 1:500 dilution of appropriate secondary conjugated antibody and 5 μg/ml Hoechst) overnight at 4°C. Embryos were washed 3×10 min in PBS-T.

The following primary antibodies were used in this study: AP2γ (RRID:AB_667770, Santa Cruz Biotechnology, sc-12762, 1:100), phosphorylated SMAD1/5/9 (a gift from Dr. Edward Laufer, University of Utah School of Medicine, USA), Sox2 (RRID:AB_11219471, Thermo Fisher Scientific, 14-9811-82, 1:200).

### Cryosectioning

Following wholemount immunostaining and imaging, embryos were oriented as desired and embedded in Tissue-Tek^®^ OCT (Sakura Finetek, Japan). Samples were frozen on dry ice for approximately 30 min and then maintained for short periods at −80°C followed by cryosectioning using a Leica CM3050S cryostat. Transverse cryosections of 10 µm thickness were cut with a Leica CM3050S cryostat and mounted on Colorfrost Plus^®^ microscope slides (Thermo Fisher Scientific) using Fluoromount G (RRID:SCR_015961, Southern Biotech, Birmingham, AL, USA). Cryosections were then imaged using a confocal microscope as described.

### Quantitative image analysis

Embryos were imaged on a Zeiss LSM880 laser scanning confocal microscope. Confocal z stacks of cells or embryo cryosections were generated. Raw data was then processed in ImageJ open source image processing software (version: 2.0.0-rc-49/1.51d). Individual PGCLCs, identified by AP2γ expression, PGCs identified by SOX2 expression, or their surrounding AP2γ− SOX2− niche cells were randomly chosen and, using Fiji (ImageJ) software, selected by manually drawing a boundary around the nucleus. The mean fluorescence intensity of pSMAD1/5/9 immunostaining, *Spry4^H2B-Venus^*, or TCF/Lef:H2B-GFP reporter expression was then measured in arbitrary units. Fluorescence decay along the *z*-axis was corrected for each channel and sample by fitting a linear regression model to the logarithm of fluorescence values as a function of the *z*-value, and correcting the models’ slopes using an empirical Bayes approach, as previously described (Saiz et al., 2016). For all quantification, statistical analysis of significance was assessed using a one-way ANOVA followed by unpaired *t*-tests to compare particular groups (GraphPad Prism, GraphPad Software, Inc., Version 7.0a). For analysis performed on embryos, all PGCs were selected from three different cryosections through the allantois of three distinct embryos. Fluorescence values were then calculated relative to the average mean fluorescence of non-neighboring (‘Other’) AP2γ− SOX2− niche cells within each individual section in order to normalize for differences in immunostaining that may arise due to differences in permeability within different embryonic regions or different stages of development. Statistics were carried out on average fluorescence levels per embryo, rather than on a per cell basis.
